# Potential Mechanisms of the Impact of Hepatocyte Growth Factor Gene-Modified Tendon Stem Cells on Tendon Healing

**DOI:** 10.3389/fcell.2021.659389

**Published:** 2021-06-18

**Authors:** Mingzhao Zhang, Hengchen Liu, Manyu Shi, Tingting Zhang, Wenjun Lu, Shulong Yang, Qingbo Cui, Zhaozhu Li

**Affiliations:** Department of Pediatric Surgery, The Second Affiliated Hospital of Harbin Medical University, Harbin, China

**Keywords:** tendon stem cells, hepatocyte growth factor, inflammation, fibrosis, tendon healing

## Abstract

The therapeutic impact of stem cells is potentially largely attributable to secretion of exosomes and soluble factors. The present study evaluates the impact of hepatocyte growth factor (HGF)–expressing tendon stem cells (TSCs) on tendon healing in a rat model. Patellar tendon TSCs were isolated and underwent transfection with lentiviral vectors containing HGF or green fluorescent protein (GFP) genes. *In vivo*, immunohistochemistry of tendons sampled 1 week postsurgery demonstrated that all stem cell–treated groups exhibited higher numbers of CD163^+^ M2 monocytes and IL-10^+^ cells (anti-inflammatory), and lower numbers of CCR7^+^ M1 monocytes and IL-6^+^ as well as COX-2^+^ cells (pro-inflammatory). Effects were most pronounced in the HGF-expressing TSCs (TSCs + HGF) treated group. Histology ± immunohistochemistry of tendons sampled 4 and 8 weeks postsurgery demonstrated that all stem cell–treated groups exhibited more ordered collagen fiber arrangement and lower levels of COLIII, α-SMA, TGF-β1, and fibronectin (proteins relevant to fibroscarring). Effects were most pronounced in the TSCs + HGF–treated group. For the *in vitro* study, isolated tendon fibroblasts pretreated with TGF-β1 to mimic the *in vivo* microenvironment of tendon injury were indirectly cocultured with TSCs, TSCs + GFP, or TSCs + HGF using a transwell system. Western blotting demonstrated that all stem cell types decreased TGF-β1-induced increases in fibroblast levels of COX-2, COLIII, and α-SMA, concomitant with decreased activation of major TGF-β1 signaling pathways (p38 MAPK, ERK1/2, but not Smad2/3). This effect was most pronounced for TSCs + HGF, which also decreased the TGF-β1-induced increase in activation of the Smad2/3 signaling pathway. The presence of specific inhibitors of these pathways during fibroblast TGF-β1 stimulation also attenuated increases in levels of COX-2, COLIII, and α-SMA. In conclusion, TSCs + HGF, which exhibit HGF overexpression, may promoting tendon healing via decreasing inflammation and fibrosis, perhaps partly via inhibiting TGF-β1-induced signaling. These findings identify a novel potential therapeutic strategy for tendon injuries, warranting additional research.

## Graphical Abstract

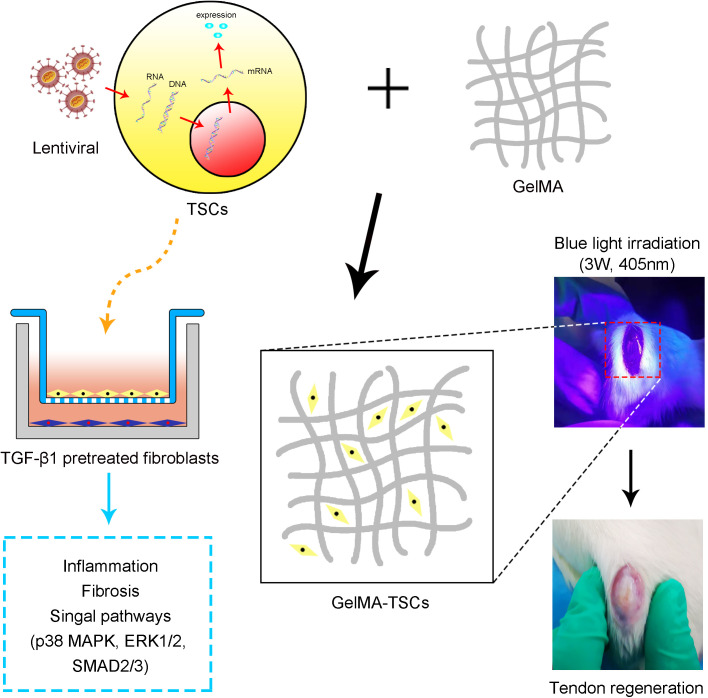

## Introduction

Tendon-related disorders and injuries are a common clinical challenge that may cause severe pain and decreased mobility; they have a significant negative impact on domains of function and quality of life and necessitate approximately 30 million surgeries per year (worldwide) (Wu et al., 2017; Rinoldi et al., 2019; Yu et al., 2020). Natural tendon healing is a slow and complex process due to tissue hypocellularity and hypovascularity (Kokubu et al., 2020). The initial response to injury is inflammation, which can result in local scar formation (Wang et al., 2019). This compromises tendon strength and elasticity, increasing the risk of damage in response to subsequent tension (Cui et al., 2011). Although current surgical and rehabilitative methods have achieved superior efficacy relative to those available in the past (Ricchetti et al., 2008; Wu et al., 2017), truly functional tendon healing remains an intractable problem requiring more advanced therapeutic strategies (Yu et al., 2020).

In recent years, the therapeutic application of tendon stem cells (TSCs) in the context of tendon injury has gradually attracted increasing attention. Such stem cells retain the potential for self-renewal and differentiation. Cumulative research demonstrates that TSCs exhibit potential in the context of tendon injury therapeutics (Zhang et al., 2011; Ni et al., 2012; Yang et al., 2017). The therapeutic effect of stem cells is attributable largely to the secretion of exosomes and soluble growth factors (Wang L. S. et al., 2017). Therefore, increasing stem cell production of beneficial cytokines and trophic factors such as IL-10, hepatocyte growth factor (HGF), and vascular endothelial growth factor (VEGF) may enhance their therapeutic paracrine wound-healing capacity (Ricchetti et al., 2008; Li et al., 2015; Wang L. S. et al., 2017; Yang et al., 2018).

The trophic factor HGF has important paracrine activities, including stimulation of cell proliferation, migration, and differentiation as well as exhibiting anti-inflammatory, anti-apoptotic and antifibrotic properties (Ricchetti et al., 2008; Wang L. S. et al., 2017). It also plays an important role in the repair of many key organs and tissues, including in the contexts of corneal injury (Omoto et al., 2017); inflammatory responses to traumatic oral ulcers (Wang X. et al., 2017); and fibrosis of the liver, kidney, and lung (Yi et al., 2014). One of our prior studies demonstrates that HGF is able to oppose scar formation by inhibiting myofibroblast differentiation and excessive extracellular matrix (ECM) deposition (Cui et al., 2011). Although application of HGF is considered effective in treating tissue injury, sustained HGF delivery to the site of injury remains a major challenge (Li et al., 2015).

The present study investigates the potential of TSCs genetically modified to achieve HGF overexpression (TSCs + HGF) to enhance tendon healing (including partially elucidating underlying mechanisms). These novel results add to the body of literature concerning improved therapeutic tendon healing strategies.

## Materials and Methods

### Isolation, Culture, and Identification of TSCs and Tendon Fibroblasts

Eight adult male Sprague–Dawley rats weighing between 180 and 200 g were used. Experimental protocols were approved by the Harbin Medical University Ethics Committee (No. Ky2018-135), and the experiment proceeded in accordance with institutional guidelines for the care and handling of laboratory animals. Isolation and identification of rat TSCs was performed as previously described (Zhang et al., 2020). Briefly, for flow cytometry, 4 × 10^6^ cells from passages three to five were used to detect those positive for surface markers CD44 and CD90, and CD11b and CD106 were used as negative markers to exclude contamination by other cells. In addition, the multilineage differentiation capacity of TSCs was assessed via staining with Oil Red O (for adipogenic potential), Alizarin Red (for osteogenic potential), and Alcian Blue (for chondrogenic potential). Rat tendon fibroblast isolation and culture was performed as previously described (Fu et al., 2008; Chang et al., 2010). Passage three to five fibroblasts were used.

### Lentiviral Transfection of TSCs

Lentiviral vectors expressing HGF (Lv-HGF, MOI = 20) and control lentiviral vectors expressing GFP (Lv-GFP, MOI = 20) were used to transfect TSCs (Hanbio, Shanghai, China); 5 × 10^5^ cells were incubated in serum-free Dulbeccos’ modified Eagle’s medium (Gibco, Invitrogen, NY, Invitrogen Corporation, Grand Island, United States) containing lentiviral vectors and 6 μg/ml polybrene (Hanbio, Shanghai, China) for 24 h at 37°C in an atmosphere containing 5% CO_2_. Thereafter, vector-containing medium was replaced with complete medium, and cells were cultured under identical conditions for a further 48 h. Successfully transfected cells were identified via fluorescence microscopy. Addition of 1 μg/ml puromycin (Thermo Fisher Scientific) for 2 to 3 days excluded untransfected cells. Expression of HGF by puromycin-resistant TSCs was further confirmed using fluorescence microscopy and Western blotting.

### Surgical Tendon Injury and Experimental Intervention

**FIGURE 1:**
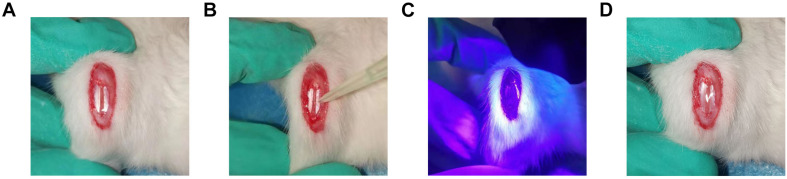
Establishment of patellar tendon injury model. **(A)** The central third of the patellar tendon was removed, resulting in a tendon defect. **(B)** GelMA containing stem cells was infused into the Patellar tendon defect. **(C)** Radiation with a 405-nm light source for 10–20 s at a distance of 3 cm from the tendon defect. **(D)** Cross-linking to form the gel state.

A total of 75 adult male Sprague–Dawley rats weighing between 180 and 200 g underwent surgery using methods similar to those previously described (Shi et al., 2019). Briefly, a third of the central part of the patellar tendon (0.8 mm in width) was removed using a surgical blade, producing a central defect. Rats were randomly assigned to five intervention groups (*n* = 15 per group): (1) TSCs + HGF, (2) TSCs + GFP, (3) TSCs (untransfected control), (4) vehicle only control, and (5) untreated control. A total of 1 × 10^6^ TSCs + HGF, TSCs + GFP, or TSCs were mixed with the vehicle, gelatin methacryloyl (GelMA) (EFL-GM-60; Suzhou Intelligent Manufacturing Research Institute, Suzhou, China). For treated groups, 30 μl of the cell-vehicle mixture was placed in the tendon defect and converted to a gel state via blue light irradiation (3 W, 405 nm) for 10–20 s with the light source placed at a distance of 3 cm from the defect. The entire remodeling process is shown in Figure 1. At 1, 4, and 8 weeks following surgery and intervention, tendon samples (*n* = 5 per time point) were collected from each group for histological evaluation and immunostaining.

### Histology and Immunostaining

Patellar tendon samples were fixed in 4% formaldehyde and embedded in paraffin. Specimens from the 4- and 8-week time points were sectioned longitudinally into 5-μm slices and stained with hematoxylin and eosin (H&E). Immunohistochemistry was performed as described previously (Wang et al., 2019). Briefly, after deparaffinization and antigen retrieval, sections were incubated with 3% H_2_O_2_ for 15 min, and 5% normal goat serum was used to block non-specific binding sites. Sections were then incubated overnight at 4°C with the following antirat primary antibodies (all Abcam, Shanghai, China): mouse anticollagen III (COLIII; monoclonal; 1:200 dilution), rabbit anti-α-smooth muscle actin (α-SMA; polyclonal; 1:200 dilution), rabbit antitransforming growth factor-β1 (TGF-β1; polyclonal; 1:500 dilution), and mouse antifibronectin (monoclonal; 1:200 dilution). Sections were then incubated with horseradish peroxidase (HRP)-conjugated secondary antibodies (Zsjqbio, Beijing, China) for 30 min at room temperature. After addition of the HRP substrate 3,3′-diamniobenzidine (DAB) and hematoxylin staining, sections were dehydrated and mounted.

Specimens from the week 1 time point underwent immunostaining for inflammatory markers as described previously (Shi et al., 2019; Wang et al., 2019). Briefly, a cryomicrotome was used to produce serial 8-μm-thick sections. Sections were incubated with goat serum for 15 min at room temperature to block non-specific binding sites, followed by incubation overnight at 4°C with the following antirat primary antibodies: rabbit anti-CD163 (monoclonal; Abcam; 1:100 dilution), rabbit anti-C-C chemokine receptor type 7 (CCR7; polyclonal; Proteintech; 1:200 dilution), rat anti-IL-10; (monoclonal; Abcam; 1:100 dilution), mouse anti-IL-6 (monoclonal; Origene; 1:100 dilution), and rat anti-cyclooxygenase-2 (COX-2; monoclonal; Abcam; 1:100 dilution). Sections were then incubated for 60 min at room temperature, in the dark, with a species-appropriate secondary antibody (Proteintech; 1:200 dilution). Cell nuclei were stained using 4′,6-diamidino-2-phenylindole (DAPI; Beyotime, Shanghai, China) for 10 min at room temperature. Finally, images were acquired using a BX53 microscope (Olympus, Japan) prior to analysis using ImageJ software.

### Stem Cell Coculture With Tendon Fibroblasts

Fibroblasts were initially precultured in medium in the presence or absence (negative control) of 5 ng/ml TGF-β1 (PeproTech, Seoul, South Korea) for 24 h at 37°C in an atmosphere containing 5% CO_2_. Then, stem cells (TSCs, TSCs + GFP, or TSCs + HGF) and tendon fibroblasts were cocultured in a transwell system (pore size: 0.4 mm) (Corning Inc., NY, United States) as described previously (Chen et al., 2018). Briefly, 1.5 × 10^5^ tendon fibroblasts in 2 ml medium were seeded into the lower chamber of each well of a six-well transwell plate, and an equivalent number of stem cells in 1 ml medium were seeded into the corresponding upper chambers. Coculture proceeded for 24 h under incubation conditions identical to those described above. To study the impact of stem cell–released soluble factors on TGF-β1-induced signaling pathways (p38 MAPK, ERK1/2, and Smad2/3) and subsequent inflammation and fibrosis, lower-chamber fibroblasts were pretreated with 20 nM each of inhibitors of p38 MAPK (SB203580), ERK1/2 (PD98059), and Smad2/3 (SB431542) activation (all MedChemExpress, CA, United States) for 1 h at 37°C prior to addition of TGF-β1.

### Protein Extraction and Western Blotting

Here, 5 × 10^5^ treated fibroblasts per group were lysed using RIPA buffer (Beyotime, Shanghai, China). The lysate was centrifuged at 12,000 rpm for 20 min at 4°C to pellet debris, and the supernatant was collected. Total protein concentration was estimated using the bicinchoninic acid (BCA) assay (Beyotime, Shanghai, China). Per sample, 10 μg of total protein was electrophoretically separated on a 10% sodium dodecyl sulfate (SDS)-polyacrylamide gel, and protein spots were transferred to a polyvinylidene fluoride (PVDF) membrane. The membrane was incubated with 5% skim milk powder in TBST for 1 h at room temperature to block non-specific binding sites, followed by incubation overnight at 4°C in the presence of antirat primary antibodies: rat anti-COX-2 (monoclonal; Abcam; 1:1000 dilution), mouse anti-COLIII (monoclonal; Abcam; 1:1000 dilution), rabbit anti-α-SMA (polyclonal; Abcam; 1:1000 dilution), anti-p38 MAPK (monoclonal; Cell Signaling Technology; 1:1000 dilution), anti-phospho-p38 MAPK (monoclonal; Cell Signaling Technology; 1:1000 dilution), anti-ERK1/2 (monoclonal; Cell Signaling Technology; 1:1000 dilution), anti-phospho-ERK1/2 (monoclonal; Cell Signaling Technology; 1:2000 dilution), anti-Smad2/3 (monoclonal; Cell Signaling Technology; 1:1000 dilution), and anti-phospho-Smad2/3 (monoclonal; Cell Signaling Technology; 1:2000 dilution). The proteins COX-2, α-SMA, and ColIII were selected as proxies to study the effects of stem cells on TGF-β1-induced inflammation and fibrosis, and the proteins p38 MAPK, ERK1/2, and Smad2/3 are involved in signaling pathways downstream of TGF-β1 that may be impacted by stem cells. Finally, the membrane was incubated with HRP-conjugated secondary antibodies for 1 h at room temperature (Boster, Wuhan, China; 1:5000 dilution) prior to addition of DAB. The membrane was imaged using a chemiluminescence CCD imaging system (ChemiScope 6200T imager, Clinx Science Instruments Co. Ltd., Shanghai, China) and protein band gray values were analyzed using ImageJ software.

### Statistical Analysis

All values are expressed as mean ± SD. Quantitative data across all groups were analyzed using one-way ANOVA followed by Tukey’s test. A *P*-value < 0.05 was considered to be statistically significant.

## Results

### Determination of TSC Phenotypes and Lentiviral Vector Transduction Efficiency

**FIGURE 2:**
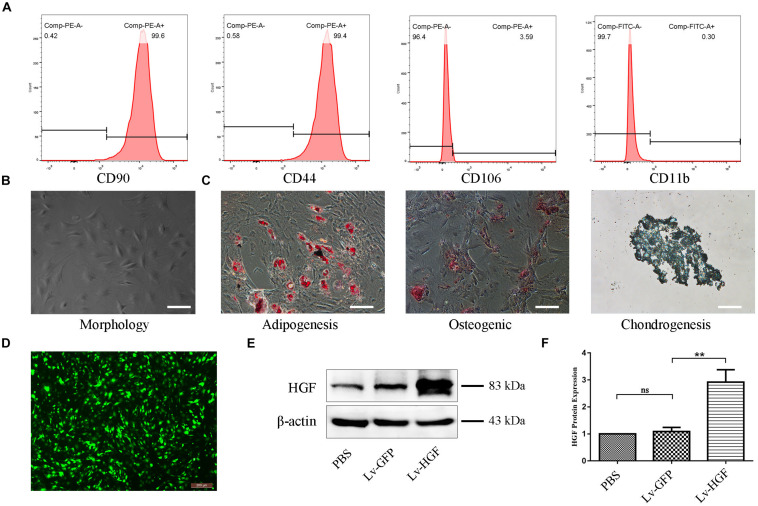
Confirmation of TSC identity, differentiation capacity, and transduction efficiency. **(A)** Flow cytometric detection of cell surface markers, including CD90, CD44, CD106, and CD11b/c. **(B)** Typical TSC morphology. **(C)** Oil Red O, Alizarin Red, and Alcian Blue stains demonstrating TSC multilineage differentiation capacity. Scale bars: 100 μm. **(D)** Fluorescent photomicrograph demonstrating examples of TSC morphology after HGF transduction and puromycin screening. **(E,F)** Western blot confirming HGF protein expression by TSCs following transduction. Data are expressed as means ± SD of three independent experiments; **P < 0.01.

Flow cytometry demonstrated that isolated TSCs were CD44^+^ CD90^+^ CD11b^–^ CD106^–^ (Figure 2A). Microscopy demonstrated typical TSC spindle-shaped morphology (Figure 2B), and cells retained multilineage differentiation capacity (ability to differentiate into adipogenic, osteogenic, or chondrogenic precursors) (Figure 2C). After TSC lentiviral (Lv-HGF, Lv-GFP) transduction and puromy1cin screening, green fluorescence demonstrated successful transduction (Figure 2D), and Western blot assessment of transduction efficiency demonstrated higher HGF protein expression in the Lv-HGF (relative to the Lv-GFP) group, but no significant difference between the PBS-only and Lv-GFP groups (Figures 2E,F).

### Effect of TSCs + HGF on the Early Inflammatory Response

**FIGURE 3:**
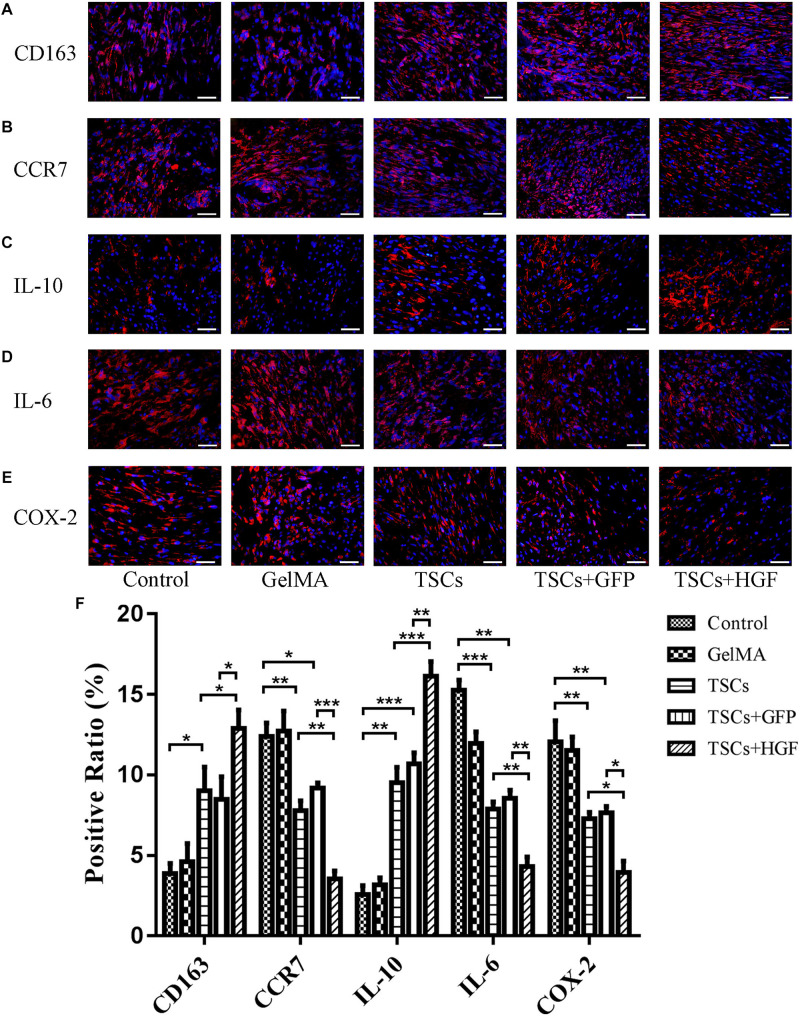
Impact of TSCs + HGF on tendon inflammation. Immunohistochemical representative images of **(A)** CD163^+^ cells, **(B)** CCR7^+^ cells, **(C)** IL-10^+^ cells, **(D)** IL-6^+^ cells, and **(E)** COX-2^+^ cells at 1 week postoperative repair site. **(F)** Positive cell ratio of inflammation-related factors at 1 week (*n* = 5). Bars: 50 μm. Data are represented as mean ± SD. *P < 0.05, **P < 0.01, ***P < 0.001.

In tendon specimens collected at week 1 postsurgery, numbers of CD163^+^ M2 macrophages (anti-inflammatory) within the defects of all stem cell–treated animals were elevated (relative to the vehicle-only and untreated control groups). This effect was most pronounced in the TSCs + HGF–treated group (Figure 3A), which also exhibited the lowest numbers of CCR7^+^ M1 macrophages (pro-inflammatory) (Figure 3B). Furthermore, the TSCs + HGF group exhibited the most significantly elevated number of IL-10^+^ (M2-favoring) and decreased number of IL-6^+^ (M1-favoring) and COX-2^+^ (prostaglandin synthesis-competent) cells, compared with other treatment groups (Figures 3C–E). Quantitative analysis results are shown in Figure 3F.

### The TSCs + HGF Group Demonstrates Improves Tendon Healing via Regulation of Tendon Matrix Deposition

**FIGURE 4:**
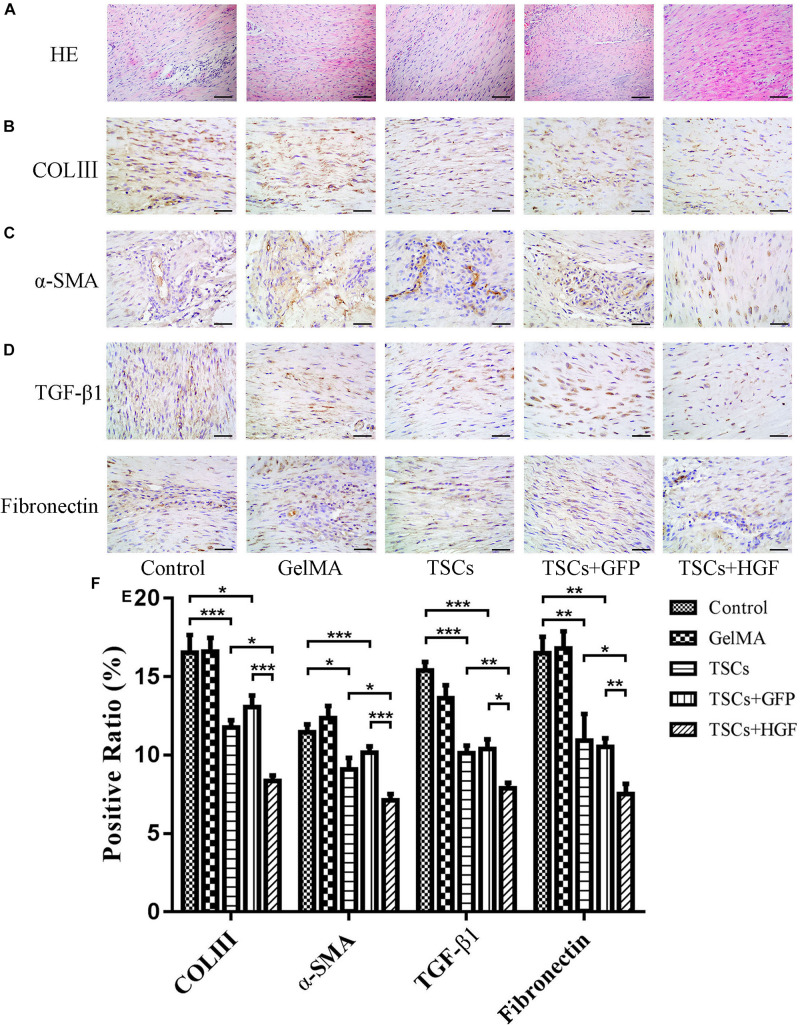
Impact of treatment with TSCs expressing HGF (TSCs + HGF) on tendon levels of fibrosis-relevant proteins at 4 weeks postsurgery. **(A)** H&E stain of the tendon repair site. Immunohistochemical evaluation of **(B)** COLIII, **(C)** α-SMA, **(D)** TGF-β1, and **(E)** fibronectin patterns. **(F)** Quantitation of fibrosis-relevant proteins (*n* = 5). Bars: 50 μm. Data are represented as mean ± SD. *P < 0.05, **P < 0.01, ***P < 0.001.

**FIGURE 5:**
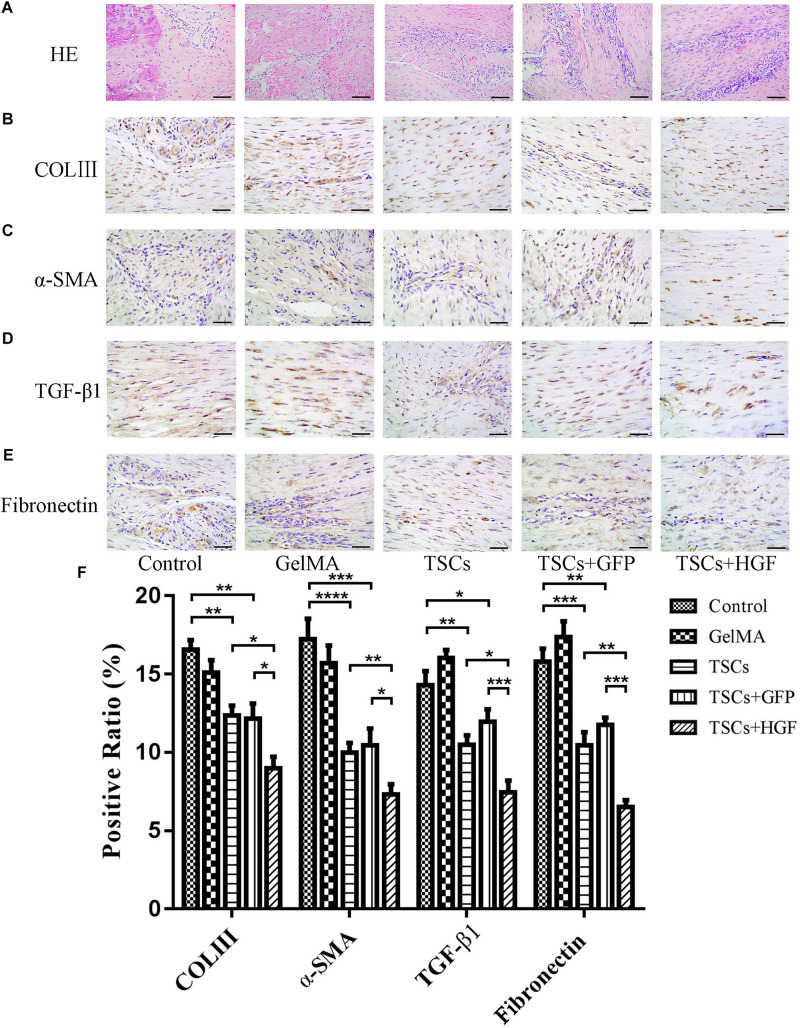
Impact of treatment with TSCs expressing HGF (TSCs + HGF) on tendon levels of fibrosis-relevant proteins at 8 weeks postsurgery. **(A)** H&E stain of the tendon repair site. Immunohistochemical evaluation of **(B)** COLIII, **(C)** α-SMA, **(D)** TGF-β1, and **(E)** fibronectin patterns. **(F)** Quantitation of fibrosis-relevant proteins (*n* = 5). Bars: 50 μm. Data are represented as mean ± SD. *P < 0.05, **P < 0.01, ***P < 0.001.

Staining of weeks 4 and 8 tendon specimens with H&E demonstrated that TSCs + HGF treatment group specimens exhibited more continuous and regular collagen fiber arrangement (relative to that of the TSCs or TSCs + GFP treatment groups) (Figures 4A, 5A). Furthermore, all stem cell–treated groups exhibited decreased levels of the tendon matrix-influencing factors COLIII, α-SMA, TGF-β1, and fibronectin at weeks 4 and 8 postsurgery, and this effect was most pronounced in the TSCs + HGF group (Figures 4B–E, 5B–E). Quantitative analysis results are shown in Figures 4F, 5F.

### Exposure to TGF-β1 Induces Inflammation and Fibrosis via Activation of Fibroblast p38 MAPK, ERK1/2, and Smad2/3 Signaling Pathways

**FIGURE 6:**
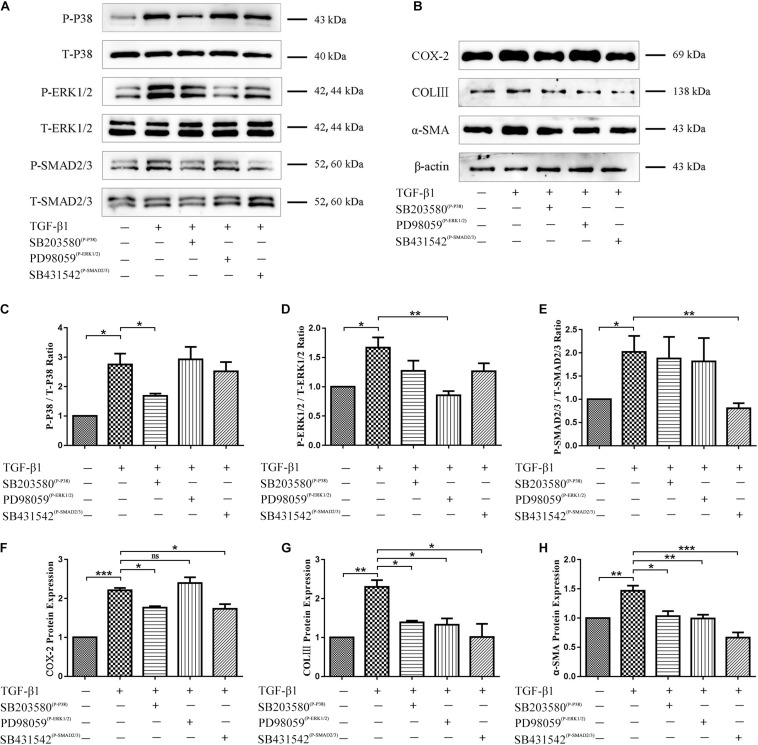
Role of p38 MAPK, ERK1/2, and Smad2/3 signaling in TGF-β1-induced inflammatory and fibrotic fibroblast functions. **(A)** Western blots confirming inhibition of p38 MAPK, ERK1/2, and Smad2/3 signaling pathways by SB203580, PD98059, and SB431542. **(B)** Western blots demonstrating levels of COX-2, COLIII, and α-SMA after inhibition of p38 MAPK, ERK1/2, and Smad2/3 signaling pathways. Quantitation of **(C)** phospho-p38 MAPK, **(D)** phospho-ERK1/2, **(E)** phospho-Smad2/3, **(F)** COX-2, **(G)** COL III, **(H)** α-SMA proportions (*n* = 3). Data are represented as mean ± SD. *P < 0.05, **P < 0.01, ***P < 0.001.

Inhibitors of p38 MAPK, ERK1/2, and Smad2/3 activation were used to determine whether these signaling pathways are involved in TGF-β1-induced fibroblast functional changes. Western blots confirmed that TGF-β1-induced activation of p38 MAPK, ERK1/2, and Smad2/3 signaling pathways was indeed inhibited (Figures 6A, C–E). Functionally, inhibition of p38 MAPK and Smad2/3 signaling co-occurred with a significant decrease in TGF-β1-induced COX-2, COLIII, and α-SMA level elevation, and inhibition of ERK1/2 signaling co-occurred only with a significant decrease in TGF-β1-induced α-SMA and COLIII (but not COX-2) level elevation (Figures 6B, F–H). These findings suggest that ERK1/2 signaling may be dispensable for TGF-β1-induced inflammation.

### Coculture With TSCs + HGF Results in Decreased Levels of COX-2, α-SMA, COLIII, p-p38 MAPK, p-ERK1/2, and p-Smad2/3 in TGF-β1-Stimulated Fibroblasts

**FIGURE 7:**
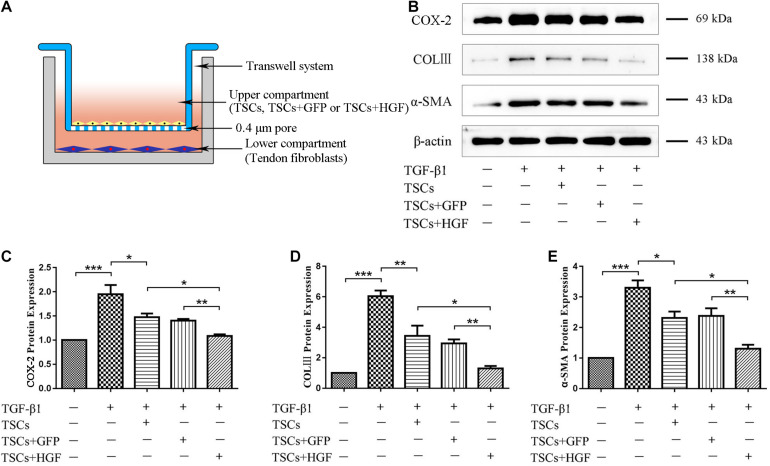
TSC and tendon fibroblast coculture system and levels of fibroblast inflammatory and fibrotic proteins. **(A)** An indirect coculture model system. **(B)** WB images of COX-2, COLIII, and α-SMA. Quantitative analysis of **(C)** COX-2, **(D)** COL III, and **(E)** α-SMA (*n* = 3). Data are represented as mean ± SD. *P < 0.05, **P < 0.01, ***P < 0.001.

**FIGURE 8:**
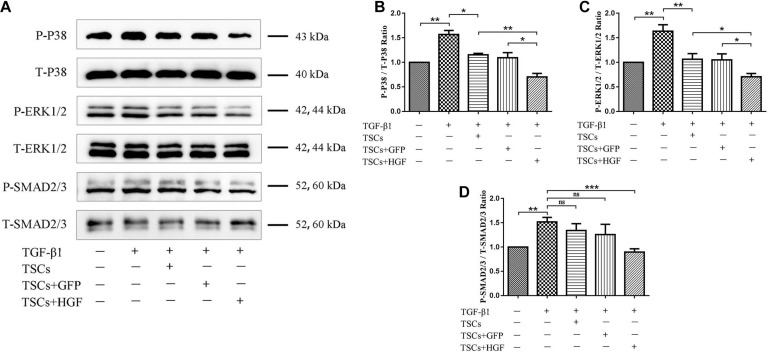
Effect of coculture with TSCs on tendon fibroblast TGF-β1-induced signaling pathways. **(A)** Western blots demonstrating p38 MAPK, ERK1/2, and Smad2/3 protein levels. Quantitation of **(B)** phospho-p38 MAPK, **(C)** phospho-ERK1/2, and **(D)** phospho-Smad2/3 proportions (*n* = 3). Data are represented as mean ± SD. *P < 0.05, **P < 0.01, ***P < 0.001.

Following indirect coculture of stem cell groups with tendon fibroblasts (Figure 7A), Western blots demonstrated that TGF-β1 increased levels of COX-2, α-SMA, and COLIII, but that all stem cell groups mitigated this effect with the most pronounced impact by TSCs + HGF (Figures 7B–E). Furthermore, TGF-β1 significantly increased the proportion of phosphorylated p38 MAPK, ERK1/2, and Smad2/3 in fibroblasts, and coculture with TSCs or TSCs + GFP inhibited these increases for p38 MAPK and ERK1/2 only, and coculture with TSCs + HGF inhibited these increases for all three proteins (p38 MAPK, ERK1/2, and Smad2/3) (Figures 8A–D). The effects of TSCs + HGF were most pronounced (Figure 8). These findings suggest that all stem cell groups (but especially TSCs + HGF) were able to decrease signaling downstream of TGF-β1 and that anti-inflammatory and antifibrotic stem cell effects may, thus, be at least partially mediated via this mechanism.

## Discussion

At present, accumulating evidence indicates that stem cells promote tissue healing mainly via paracrine mechanisms (Zhao et al., 2018). One important trophic factor with paracrine activity, HGF, exhibits a variety of functions, including promoting angiogenesis, regulating inflammation, inhibiting fibrosis, and promoting tissue regeneration (Wang L. S. et al., 2017). Our previous study also reveals that the use of exogenous HGF has a positive therapeutic effect on the formation of fibrosis and scar in tendon injury healing (Cui et al., 2011). We, therefore, hypothesized that tendon healing may be enhanced via sustained TSC expression of HGF.

Tendon healing occurs in three stages: inflammation, cell proliferation, and tissue remodeling. The present study initially examined the role of TSCs + HGF in regulating the inflammatory response *in vivo*. During recent years, the importance of macrophages and various inflammatory mediators in tendon healing has become apparent, and it is now believed that “molecular inflammation” plays a key role in tendon pathophysiology (Sunwoo et al., 2020). Macrophages exist as functionally distinct phenotypes with differential roles during tendon healing (Sunwoo et al., 2020). Classically activated macrophages (M1) secrete pro-inflammatory cytokines early during the wound-healing process, and alternatively activated macrophages (M2) promote wound healing during the inflammatory response phase by means of producing anti-inflammatory factors and antagonizing M1 responses (Saleh et al., 2019). Although M1 macrophages can cause ECM degradation, inflammation, and apoptosis (Klinkert et al., 2017; Laplante et al., 2017; Dai et al., 2018), M2 macrophages can inhibit inflammation, prevent scar formation, and enhance tendon strength (Chamberlain et al., 2019). Switching macrophages from the M1 to the M2 phenotype after tendon injury can promote tendon healing (Manning et al., 2015). The present study demonstrates that treatment with TSCs + HGF significantly increases numbers of CD163^+^ M2 macrophages and decreases numbers of CCR7^+^ M1 macrophages (more so than treatment with either TSCs or TSCs + GFP) and that levels of M2-favoring IL-10 are highest, and those of M1-favoring factor IL-6 and pro-inflammatory factor COX-2 are lowest in the TSCs + HGF treatment group. These findings suggest that TSCs + HGF have therapeutic potential to promote tendon healing via anti-inflammatory modulation of macrophages and associated cytokines.

However, inflammation is a double-edged sword. On the one hand, it prevents wound infection and can induce tissue healing; on the other hand, excessive inflammation can lead to scarring, thereby limiting tendon mobility and strength and increasing the risk of tendon reinjury (Wang et al., 2019). To investigate whether inhibiting inflammation is associated with decreased fibrosis and scar formation during tendon healing, the present study evaluated the expression of COLIII, α-SMA, TGF-β1, and fibronectin. Collagen is the major tendon constituent with parallelized COLI being the most abundant subtype. Indeed, COLI accounts for about 90% of normal tendon mass, and COLIII makes up less than 10% of this mass. A substantial increase in disorganized COLIII is typically observed in scar tissue, and decreases tendon strength while increasing the risk of reinjury (Liao et al., 2020). Previous studies demonstrate that increased COLIII expression is associated with scar formation and inferior tendon mechanical properties after healing (Matsumoto et al., 2002; Juneja et al., 2013). The present study demonstrates significantly lower COLIII levels in all stem cell–treated groups but especially in the TSCs + HGF group, suggesting a positive effect of stem cells on tendon repair. We have previously demonstrated that inhibiting overexpression of the myofibroblast marker α-SMA restrains scar formation following tendon injury (Cui et al., 2011). The present study demonstrates that α-SMA levels in tendons sampled at weeks 4 and 8 postsurgery are significantly lower in all stem cell–treated groups, but especially in the TSCs + HGF group, suggesting inhibition of myofibroblast differentiation, which may contribute to decreased scar formation. In addition, the TSCs + HGF group demonstrates the lowest levels of TGF-β1 and fibronectin. Like inflammation, TGF-β1 can have context-dependent differential effects. Although it is important during the initial stages of tissue healing (contributing to scaffold formation for local tissue outgrowth), high levels of TGF-β1 during the later stages of wound healing can lead to scarring and impaired tissue function (James et al., 2008; Cui et al., 2011). Fibronectin exists largely as an ECM component of both embryonic and adult tissues; it is also associated with the formation of scar tissue and adhesions after tendon injury and has a profound influence on wound-healing quality (Wang et al., 2019). The present findings suggest that TSCs + HGF may promote high-quality tendon healing (including inhibition of scar formation).

In recent years, GelMA has attracted attention in the domain of tissue engineering due to its excellent biological properties and experimenter ability to control its shape (including formation of three-dimensional structures) for personalized tissue repair (Yue et al., 2015; Shao et al., 2018). GelMA provides a degradable polymeric scaffold able to support cell attachment, infiltration, and proliferation; stem cell loading of GelMA has been widely explored for wound repair, and this material is proven to be an effective tissue engineering scaffold (Saleh et al., 2019; Lee et al., 2020; Luo et al., 2020). To the best of our knowledge, the present study is the first to apply TSC-loaded GelMA in a rat patellar tendon injury model. In our hands, GelMA photo-crosslinking stably attached the cell-loaded gel to the patellar tendon window defect, facilitating location-targeted cell therapy without the loss of cells. Thus, initial findings indicate that this may be a promising therapeutic strategy to enhance tendon healing.

The present study used a transwell coculture system to study the impact of TSCs + HGF on TGF-β1-induced tendon fibroblast function. As mentioned, TGF-β1 activity is implicated in fibrotic disorders and plays crucial roles in tissue healing and the pathogenesis of scarring and formation of adhesions (Yao et al., 2020). A large number of studies employ various strategies in an attempt to counter the more pathological effects of TGF-β1. For example, RelA/p65 inhibition prevents the formation of tendon adhesions by inhibiting TGF-β1-induced inflammation and cell proliferation (Chen et al., 2017); microRNA-21-3p gene-modified umbilical cord stem cell–derived exosomes inhibit tendon inflammation and adhesion formation by inhibiting TGF-β1-induced expression of COX-2, α-SMA, and COLIII (Yao et al., 2020); HGF can alleviate renal interstitial fibrosis via inhibiting the TGF-β1/SMAD pathway (Xu et al., 2018) and also regulates the activation of TGF-β1 in rat hepatocytes and hepatic stellate cells (Narmada et al., 2013); and we have previously demonstrated that HGF inhibits TGF-β1-induced fibrosis in a rat Achilles tendon injury model (Cui et al., 2011).Therefore, the present study pretreated tendon-derived fibroblasts with TGF-β1 to investigate whether the beneficial effects of stem cells on tendon healing may be associated with impacts on TGF-β1-induced pathways. Consistent with previous studies, TGF-β1 enhanced tendon fibroblast levels of inflammation and fibrosis indicators, including COX-2, α-SMA, and COLIII (Yao et al., 2020). Increased levels of α-SMA and COLIII likely indicate excessive deposition of ECM components: a pathological hallmark of fibrotic disorders. In addition, pro-inflammatory COX-2 is also associated with fibrosis and formation of adhesions after tendon injury (Chen et al., 2017; Yao et al., 2020). As hypothesized, all stem cell–exposed fibroblast groups, but especially the TSCs + HGF-exposed group, exhibited decreased TGF-β1-induced increases in indicator protein levels. These findings suggest that the beneficial effects of TSCs + HGF on tendon healing may be mediated in part via countering TGF-β1-induced functions. To further investigate the possibility that TSCs + HGF anti-inflammatory and antifibrotic effects are mediated via altered TGF-β1-induced signaling pathways, we studied stem cell impact on the major signal transduction pathways downstream of TGF-β1 (p38 MAPK, ERK1/2, and Smad2/3); these pathways are involved in cell proliferation, differentiation, development, inflammation, and apoptosis (Nuwormegbe et al., 2017). Coculture with TSCs + HGF most significantly inhibited TGF-β1-induced fibroblast increases in phosphorylated p38 MAPK, ERK1/2, and Smad2/3 protein levels, concomitant with a significant decrease in myofibroblast differentiation, and ECM synthesis. However, decreased ERK1/2 phosphorylation did not decrease levels of inflammatory indicators. These findings suggest that the antifibrotic effects of TSCs + HGF may be mediated via downmodulation of the p38 MAPK, ERK1/2, and Smad2/3 signaling pathways, and their anti-inflammatory effects may be mediated via downmodulation of the p38 MAPK and Smad2/3 (but not the ERK1/2) signaling pathways. Exposure to the other stem cell groups (TSCs and TSCs + GFP) also decreased fibroblast p38 MAPK and ERK1/2 (but not Smad2/3) pathway activation, suggesting that their therapeutic effects may be mediated via downmodulation of the p38 MAPK and ERK1/2 signaling pathways. Given that TSCs + HGF demonstrate the most pronounced effect on signaling pathway activation downmodulation, they may ultimately provide a more potent therapeutic effect than the other stem cell groups.

However, the present study suffers from several limitations. First, only a 1:1 coculture ratio of the two cell types was examined. Second, *in vitro* studies did not explore the impact of stem cells (including TSCs + HGF) on tenocytes. Third, the mechanism study of the effects of TSCs + HGF on immune cells is lacking. Finally, *in vivo* studies only show the histology by H&E staining, which may not completely detail the positive effect of TSCs + HGF on tendon repair. Further investigation may be required to prove this, including quantified histology scores, mechanical testing, and the size of collagen fibrils, among others. All these limitations are the bases and direction for further studies.

## Conclusion

Taken together, findings of the present study demonstrate that increased HGF provision (by TSCs + HGF) inhibits inflammation and scar formation during tendon healing and suggest that inhibition of TGF-β1-induced p38 MAPK and Smad2/3 signaling may contribute to anti-inflammatory effects, while inhibition of TGF-β1-induced p38 MAPK, ERK1/2, and Smad2/3 signaling may contribute to antifibrotic effects. Thus, this study provides evidence supporting HGF-overexpression in TSCs possibly as a novel therapeutic strategy to enhance tendon healing. Additional future studies are warranted.

## Data Availability Statement

The raw data supporting the conclusions of this article will be made available by the authors, without undue reservation.

## Ethics Statement

The animal study was reviewed and approved by the Harbin Medical University Ethics Committee.

## Author Contributions

MZ: experimental design, cytology experiments, animal experiments, data acquisition, data analysis, and manuscript writing. HL: cytology experiments, animal experiments, data acquisition, data analysis, and final approval of manuscript. TZ and MS: statistical analysis of the data and final approval of manuscript. WL and SY: experimental technical support and final approval of manuscript. QC and ZL: experimental design, text revision, and final approval of manuscript. All authors contributed to the article and approved the submitted version.

## Conflict of Interest

The authors declare that the research was conducted in the absence of any commercial or financial relationships that could be construed as a potential conflict of interest.
